# Appropriateness of the Empirical Antibiotics Prescribed and Their Concordance with National Guidelines for Three Selected Infections among Cancer Patients in a Tertiary Care Centre in Sri Lanka

**DOI:** 10.1155/2021/7572215

**Published:** 2021-09-28

**Authors:** Gayashan Chathuranga, Thushari Dissanayake, Neluka Fernando, Chandanie Wanigatunge

**Affiliations:** ^1^Department of Medical Laboratory Sciences, Faculty of Allied Health Sciences, University of Sri Jayewardenepura, Nugegoda, Sri Lanka; ^2^Department of Microbiology, Faculty of Medical Sciences, University of Sri Jayewardenepura, Nugegoda, Sri Lanka; ^3^Department of Pharmacology, Faculty of Medical Sciences, University of Sri Jayewardenepura, Nugegoda, Sri Lanka

## Abstract

**Background:**

Prophylactic and empirical antibiotic use is essential in cancer patients due to the underlying immune deficiencies. We examined the spectrum of causative bacteria and the appropriateness of empirical antibiotic prescription for three selected infections in cancer patients. *Methodology*. A descriptive cross-sectional study was conducted at the National Institute of Cancer (NIC), Sri Lanka, from June 2018 to February 2019. Bacterial isolates obtained from adult cancer patients with a diagnosis of lower respiratory tract infections (LRTI), skin and soft tissue infections (SSTI), or urinary tract infections (UTI) were included. Causative bacteria were identified and the antibiotic susceptibility was determined by standard microbiological methods. Empirical therapy was defined as appropriate if the isolated pathogen was susceptible in vitro to the given antibiotic.

**Results:**

A total of 155 bacterial isolates were included in the analysis. LRTI were the most prevalent infections (37.2%, 55/148) encountered during the study period. Majority (90.9%) of the isolated bacteria were ESKAPE pathogens. *Klebsiella pneumoniae* was the most frequent pathogen causing LRTI (42.4%, 25/59), whereas *Escherichia coli* (32%, 16/50) and *Staphylococcus aureus* (26.1%, 12/46) predominated in UTI and SSTI, respectively. Meropenem was the most prescribed empirical antibiotic for LRTI (29.1%, 16/55) and SSTI (26.6%, 11/43) while it was ceftazidime for UTI (36%, 18/50). Only 20.6% (32/155) of the isolated bacteria were susceptible to the empirical antibiotic prescribed while 48.4% (75/155) were resistant to them. The prescribed empirical antibiotic did not have the spectrum of activity for the isolated bacteria in 29% (45/155) of cases.

**Conclusion:**

High resistance rates were observed against the prescribed empirical antibiotics. National empirical antibiotic guidelines should be revised with updated data on causative organisms and their susceptibility patterns to ensure appropriate empirical antibiotic prescription.

## 1. Introduction

Cancer is a major cause of morbidity and mortality in the 21^st^ century. With an estimated 18.1 million new cases and 9.6 million deaths in 2018, cancer is expected to rank as the leading cause of death in every country of the world [1]. Infections increase the mortality in those with cancers and in the United States; it is the 2^nd^ leading “noncancer” cause of death among such patients [2]. In the US, approximately 60% of the deaths in patients with haematological malignancies and 50% of the deaths in patients with solid organ tumors are infection related [3, 4]. Blood stream and respiratory infections have been identified as the most frequent groups of infections among cancer patients followed by urinary tract, skin and soft tissue, and gastrointestinal infections [5, 6]. Cancer is ranked as the 2^nd^ leading cause of hospital deaths in Sri Lanka [7], but there is a lack of published data with regard to infections in cancer patients.

Early initiation of appropriate antibiotics is crucial for effective management of infections in cancer patients. Targeted antibiotic therapy is possible only when the causative pathogen is identified by cultures with determination of the antibiogram. As this usually takes 24 to 48 hours, antibiotics are often given on an empirical basis guided by the clinical presentation. A delay in antibiotic administration has shown to increase the length of hospital stay in cancer patients [8].

Empirical therapy is defined as the initial antibiotic regimen started within 24 hours of admission/first encounter with the patient [9, 10]. There are published guidelines and local recommendations (e.g., the Sanford Guide to Antimicrobial Therapy, Practice Guidelines of the Infectious Diseases Society of America, and Empirical and Prophylactic Use of Antimicrobials: National Guidelines, Sri Lanka) regarding the appropriate empirical antibiotics for infections. However, these guidelines should be periodically evaluated as the microbiological epidemiology and the antimicrobial susceptibility patterns continuously change over time. One of the main reasons for not updating the guidelines in a timely manner, especially in low-income countries, is the limited availability of local data on antibiotic use in humans [11].

The spectrum of bacterial pathogens isolated from cancer patients has changed over the past few decades. There has been a shift towards multidrug-resistant bacteria (MDR) such as extended-spectrum *β*-lactamase-producing (ESBL) *Enterobacteriaceae*, carbapenem-resistant *Enterobacteriaceae* (CRE), multidrug-resistant *Acinetobacter baumannii,* and methicillin-resistant *Staphylococcus aureus* (MRSA) [12]. However, existing guidelines do not clearly define antibiotic regimens for these MDR bacteria causing infections in cancer patients [13, 14].

Therefore, it is important to study the existing epidemiological profile and susceptibility pattern of bacterial pathogens, especially in high-risk patient groups such as cancer patients. This will help in optimizing the antibiotic prescription and thereby diminish the upward trend in antibiotic resistance [15].

There is limited information regarding the microbiological epidemiology of cancer patients in Sri Lanka. This study was conducted to assess the appropriateness of the empirical antibiotics prescribed to cancer patients for three selected infections and fill these knowledge gaps with respect to infections in patients with malignancies in the country.

## 2. Materials and Methods

This study was conducted at the National Institute of Cancer (NIC), Sri Lanka, from June 2018 to February 2019. NIC is the main tertiary-care institute in the country dedicated for cancer care.

### 2.1. Study Population

Bacterial isolates obtained from adult cancer patients (i.e., >18 years) diagnosed by the attending physicians as having lower respiratory tract infections (LRTI), skin and soft tissue infections (SSTI), or urinary tract infections (UTI) were included in the analysis.

### 2.2. Inclusion and Exclusion Criteria

Bacterial isolates from adult cancer patients presenting with the three selected infections and for whom parenteral empirical antimicrobial therapy was indicated were included in the study. As prolonged antibiotic exposure might influence the spectrum of isolated bacteria due to selective pressure [16], bacterial isolates obtained from patients who were on parenteral antibiotics for more than 48 hours before specimen collection for culture were excluded. Bacterial isolates from intensive-care patients were excluded as the infections in intensive-care patients are considered as a distinct category of infections with a predominant prevalence of multidrug-resistant bacteria (MDR) [17, 18].

Patient demographic data (age, gender, primary cancer site) and the data regarding antibiotic use (choice of empirical antibiotic, duration) were extracted from in-patient clinical records.

### 2.3. Microbiological Methods

Bacterial isolates obtained from positive cultures performed in the microbiology laboratory at the hospital were subcultured onto suitable media and incubated at 35–37°C for 24–48 hours. All culture media (blood agar base, MacConkey agar, and Mueller-Hinton agar) were purchased from Oxoid Limited, UK. Microorganisms were identified by Gram staining followed by standard biochemical methods [19]. Bacteria in the family *Enterobacteriaceae* and genus *Acinetobacter* were identified up to the species level by API® 20E and 20NE test kits (bioMérieux, USA), respectively. Antibiotic susceptibility tests (ABST) were conducted by the disk diffusion technique according to the Clinical and Laboratory Standards Institute (CLSI) 2018 guidelines [20]. Antibiotic disks were purchased from Mast Group Ltd., UK (MASTDISCS® *AST*). Selection of the antibiotics for the susceptibility testing was done according to the guidelines given by the Sri Lanka College of Microbiologists [19]. Extended-spectrum *β*-lactamase (ESBL) production in *Enterobacteriaceae* was phenotypically confirmed by combined disk diffusion test (CLSI recommended method) [20].

National guidelines on empirical use of antibiotics published by the Sri Lanka College of Microbiologists [21] were used as a guide to determine whether the empirical antibiotic prescription was in accordance with the guidelines.

The initial empirical treatment was considered to be appropriate if the subsequently isolated pathogen was found to be susceptible to the empirical antibiotic in vitro. Therapy was considered inappropriate when either the causative bacteria was resistant to the administered empirical antibiotic or when the empirical antibiotic did not have the spectrum of activity according to the Sanford Guide [22] against the causative bacteria.

### 2.4. Statistical Analysis

IBM SPSS statistics version 25 was used for data analysis. Descriptive statistics was used to calculate frequencies and proportions. Chi-square test was used to determine the significance between proportions/percentages.

## 3. Results

One hundred and fifty-five bacterial isolates from 148 cancer patients presenting with the selected infections were analyzed during the study period. Of the bacterial isolates, 62.2% (92/148) were from patients with solid tumors while 37.8% (56/148) were from patients with haematological malignancies. Acute myeloid leukaemia 37.5% (21/56) and acute lymphoblastic leukaemia 21.4% (12/56) were the commonest haematological malignancies, while oral cancer 15.2% (14/92) and colorectal cancer 15.2% (14/92) were the commonest in patients with solid tumor.

A total of 155 bacterial pathogens were isolated. Polymicrobial infections were seen in 4.7% (7/148) of the patients. LRTI were the commonest infections (37.2%, 55/148) encountered during the study period followed by UTI (33.8%, 50/148) and SSTI (29.1%, 43/148).

### 3.1. Bacterial Spectrum and Susceptibility Patterns

Gram-negative organisms accounted for 80.6% (79/98) and 78.9% (45/57) of the isolates from those with solid tumors and haematological malignancies, respectively.

**Figure 1:**
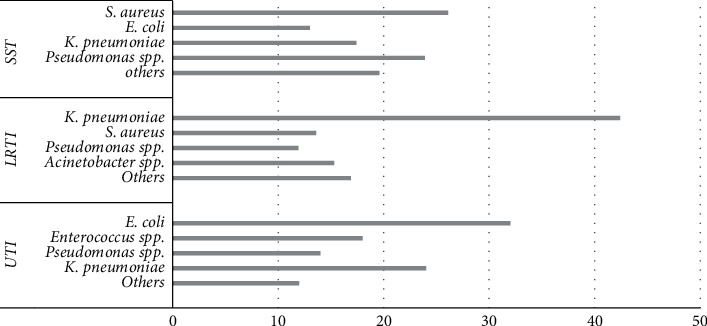
Frequency of bacterial species isolated from the patients in each group of infection. SST, skin and soft tissue infections; LRTI, lower respiratory tract infections; UTI, urinary tract infections.

*Klebsiella pneumoniae* was the most frequently isolated pathogen from LRTI (42.4%, 25/59), whereas *Escherichia coli* (32%, 16/50) and *Staphylococcus aureus* (26.1%, 12/46) predominated in UTI and SSTI, respectively (Figure 1).

*K. pneumoniae* was also the commonest bacteria (29%, 45/155) in the three types of infections followed by *E. coli* (17.4%, 27/155). Bacteria in the family *Enterobacteriaceae* were identified as the pathogen in 58.1% (90/155) of the isolates (Table 1).

Highest susceptibility of the *Enterobacteriaceae* isolated from all three groups of infections was observed for amikacin while the lowest susceptibility was observed for amoxicillin-clavulanic acid (Table 2). Extended-spectrum *β*-lactamase production was detected in 20% (18/90) of the *Enterobacteriaceae* isolates and carbapenem resistance was found to be 41.1% (37/90). Carbapenem resistance among *K. pneumoniae* (54.2%) was significantly higher than that of the other coliforms (26.2%) (*p*=0.007). Isolates of *Pseudomonas* spp. demonstrated the highest susceptibility for amikacin (72%, 18/25) and gentamicin (68%, 17/25) while the lowest susceptibility was observed for ticarcillin-clavulanic acid (24%, 6/25).

In our samples, *Staphylococcus aureus* isolation rate was 13.5% (21/155) and 71.4% (15/21) of those were methicillin resistant (MRSA). Inducible clindamycin resistance was detected among 19% (4/21) of *S. aureus* isolates. All *S. aureus* isolates obtained from patients with LRTI (*n* = 8) were MRSA (Table 3). *Enterococcus* species were isolated from nine patients with UTI and three (33.3%) of those were vancomycin resistant.

### 3.2. Empirical Antibiotics Prescribed

Meropenem was the most commonly prescribed empirical antibiotic for LRTI (29.1%, 16/55) and SSTI (25.6%, 11/43) while it was ceftazidime (36%, 18/50) for UTI.

**Figure 2:**
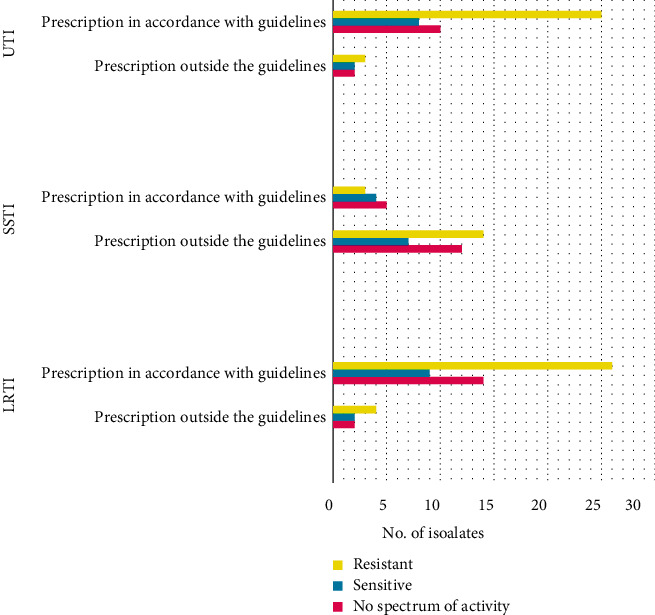
Susceptibility pattern of the isolated bacteria to the empirical antibiotics.

Prescription of empirical antibiotics for LRTI was in accordance with the national guidelines for 83.6% (46/55) of the patients (Figure 2). Resistance to prescribed antibiotics was seen with 50.8% (30/59) of the isolates. For 27.1% (16/59) of the isolates, the prescribed antibiotic did not have the spectrum of activity for the isolated bacteria. Recommended antibiotic had not been included in the ABST for 2 isolates as the particular antibiotic cannot be tested with the disk diffusion method.

Adherence to national guidelines was observed in 86% (43/50) of the empirical antibiotic prescriptions for UTI. However, 58.1% (25/43) of the isolated bacteria were resistant against these antibiotics (Figure 2). For 16.2% (7/43) of the patients, empirical therapy was in accordance with the guidelines and targeted at Gram-negative bacteria, yet the isolated bacteria were Gram-positive organisms.

Empirical antibiotic prescription for SSTI showed least concordance with the national guidelines (25.5%, 11/43). For 74.4% (32/43) of the patients, the recommended antibiotics were outside the guidelines. However, only 21.2% (*n* = 7) of the isolates were sensitive to the antibiotics prescribed outside the guidelines (Figure 2).

Overall, 48.4% (75/155) of the bacterial isolates were resistant to the recommended empirical antibiotics, while in 29% (45/155), the selected empirical antibiotic did not have the spectrum of activity for the isolated bacteria. Isolated bacteria were susceptible to the empirical antibiotics only in 20.6% (32/155) and can be considered as appropriate prescription.

## 4. Discussion

Bacterial spectrum and the susceptibility pattern of pathogenic bacteria have changed over the past few decades in cancer patients [23, 24]. In these patients, majority of the blood stream infections are caused by Gram-positive organisms, whereas Gram-negative bacteria predominate in other sites of infection [5, 25, 26]. Our study shows a predominance of Gram-negative bacteria in all three selected infections. A similar study conducted in Turkey has shown a high rate of bacteria in the family *Enterobacteriaceae* from cancer patients with infections other than from the blood stream [26]. A similar pattern was observed in our study where a higher prevalence of family *Enterobacteriaceae* was seen. In a study by Sirkhazi et al. [25], *E. coli* was the most predominant isolate obtained from cancer patients with infections. However, in our study, the commonest was *Klebsiella pneumoniae* followed by *E. coli*.

Increasing antimicrobial resistance has become a major problem in several cancer treatment facilities worldwide [27]. Emergence of ESBL-producing *Enterobacteriaceae* has become a serious problem to which there are very few treatment alternatives. Carbapenems are the corner stone of treatment for these organisms, and because of its increased usage for empirical therapy, the risk of selecting resistant organisms is increasing [25]. Our findings also showed a higher usage and a resistance rate for carbapenem in cancer patients.

In 2008, Rice [28] introduced a group of bacteria under the acronym “ESKAPE” which consist of *Enterococcus faecium*, *Staphylococcus aureus*, *Klebsiella pneumoniae*, *Acinetobacter baumannii*, *Pseudomonas aeruginosa,* and *Enterobacter* spp. These organisms were identified as important pathogens for nosocomial infections with potential antibiotic resistance. Recently, several studies have suggested adding *E. coli* to the ESKAPE group with regard to the infections in immunocompromised patients [29, 30]. Proportion of ESKAPE bacteria including *E. coli* isolates in our study was 90.8%. Bodro et al. have shown that the cancer patients infected with drug-resistant ESKAPE pathogens (rESKAPE) often receive inappropriate empirical antibiotic therapy [29]. This was clearly observed in our study with nearly 50% bacteria resistant to empirical therapy.

A comparable resistance to amoxicillin-clavulanic acid in the *Enterobacteriaceae* family as stated by Fentie et al. [31] was seen in our study. Although Sirkhazi et al. [25] and Zorgani et al. [32] have observed nearly 100% sensitivity for carbapenems among *Enterobacteriaceae,* a high resistance (41.1%) was seen in our study. ESBL production was phenotypically confirmed only in 18 *Enterobacteriaceae* isolates (20%) and all those isolates were sensitive to carbapenems. However, all the carbapenem-resistant *Enterobacteriaceae* isolates were resistant to 3^rd^ generation cephalosporins. This could be due to these isolates harbouring other coexisting resistance mechanisms such as Amp-C production [33]. Coexistence of such resistance mechanisms can impede the detection of ESBL by commonly used phenotypic tests, thus giving rise to a falsely low ESBL rate by these detection methods.

The higher rates of carbapenem resistance (41.1%) and MRSA (71.4%) seen in our study compared to other similar studies [25, 31, 34] is a cause for concern. While this indicates changing patterns of antibiotic resistance, it needs further investigation to identify the reasons and measures to curtail it.

A relatively high adherence to national guidelines was noted with nearly 70% of patients' empirical antibiotic prescriptions which were compatible with the guideline. This was comparable to a study done in the United States, where the use of appropriate empirical antibiotic therapy was high with more than 80% of cancer patients admitted with febrile neutropenia receiving guideline-concordant antibiotics [35]. However, despite this high concordance with national guidelines, only 20.6% of the patients had received appropriate empirical therapy, as nearly 50% of the isolates were resistant to these antibiotics in vitro. This is similar to the results of a study conducted among oncohematological patients with febrile neutropenia in Spain which revealed the inadequacy of empirical therapy despite the high adherence to guidelines [36].

To the best of our knowledge, this is the first comprehensive study to investigate the microbial spectrum and antibiotic sensitivity pattern of bacterial pathogens causing LRTI, SSTI, and UTI in cancer patients in Sri Lanka. As we also determined the antibiotics prescribed for this patient population, it provides information helpful to identify the relationship between antibiotic usage and development of resistance. Although the study was done in a single centre, the findings are likely to reflect the ABST patterns seen in Sri Lanka, as the centre receives patients from all over the country.

Antimicrobial prescribing guidelines and formularies aim to promote responsible prescribing that maximizes benefit to the patient while minimizing the emergence of resistant microorganisms. Although international committees have published evidence-based guidelines to guide the choice of appropriate empiric antibiotic regimens, it is still unclear whether they are optimal locally, where antimicrobial sensitivity and resistance patterns may differ. Therefore, it is important to develop national guidelines to meet local needs. The current Sri Lankan guidelines on empirical antibiotic therapy were published in 2016 and a revision of this is urgently needed to address the changing patterns of antibiotics sensitivity of bacteria. The high resistance seen for recommended empirical antibiotics in populations such as cancer patients suggests that special considerations should be made for these categories. The empirical use of broad-spectrum antimicrobial regimen, especially for coverage of Gram-negative bacteria must also be considered.

Despite being a developing country with limited resources, Sri Lanka still provides free healthcare to its population. Yet, many areas need improvement. National policies to strengthen and optimize antibiotic stewardship programmes are urgently needed to improve antibiotic use and minimize the emergence of resistant organisms. This, in turn, would help to reduce healthcare costs to both the individual patients and the country.

## 5. Conclusion

In conclusion, our study shows similar results with other studies in overall prevalence of Gram-negative bacteria in the selected infection groups. Majority of the isolated bacteria were ESKAPE pathogens. High resistance rates against most of the antibiotics tested were observed, particularly among coliforms which might limit the choice of empirical antibiotics. A considerable proportion of isolated bacteria in this study showed resistance against the antibiotics recommended in the national empirical guidelines. Continuous surveillance is essential to update the existing guidelines as pathogens and susceptibility patterns change over time.

## Figures and Tables

**Table 1:** Frequency of *Enterobacteriaceae* (coliforms) isolated from the patients.

	LRTI	SSTI	UTI	Total
*Klebsiella pneumoniae*	25	08	12	45
*Escherichia coli*	05	06	16	27
*Proteus mirabilis*	—	05	02	07
*Klebsiella oxytoca*	01	01	01	03
*Enterobacter cloacae*	01	02	—	03
*Enterobacter aerogenes*	02	—	—	02
*Morganella morganii*	—	—	01	01
*Providencia stuartii*	—	01	—	01
*Shigella* species	—	—	01	01
Total frequency				90 (58.1%, 90/155)

**Table 2:** Susceptible proportions of *Enterobacteriaceae* (coliforms) in each infection group.

	Susceptible proportion (%)		
	LRTI (*n* = 34)	SSTI (*n* = 23)	UTI (*n* = 33)
Ciprofloxacin	14.7	13.0	15.2
Co-trimoxazole	^*∗∗*^	26.1	30.3
Co-amoxiclav	2.9	4.3	9.1
Gentamycin	73.5	30.4	48.5
Cefuroxime	2.9	13	18.2
Amikacin	73.5	65.2	81.8
Imipenem/meropenem	47.1	60.9	60.6
Piperacillin-tazobactam	23.5	52.2	30.3
Ticarcillin-clavulanic acid	11.8	^*∗∗*^	15.2
Cefotaxime	14.2	13.0	18.2
Aztreonam	17.6	13.0	27.3
Nitrofurantoin	^*∗∗*^	^*∗∗*^	48.5
Nalidixic acid	^*∗∗*^	^*∗∗*^	6.1
Norfloxacin	^*∗∗*^	^*∗∗*^	18.2
ESBL production	8.8 (3/34)	34.8 (8/23)	21.2 (7/33)
Carbapenem resistance	50 (17/34)	38.8 (8/23)	36.4 (12/33)

^*∗∗*^Not tested as these are not indicated for the treatment of selected infections.

**Table 3:** Susceptible proportions of isolated *Staphylococcus aureus*.

	Susceptible proportion (%)	
	SSTI (*n* = 12)	LRTI (*n* = 8)
Cloxacillin	58.3	00
Ciprofloxacin	16.7	25
Erythromycin	41.7	12.5
Clindamycin	58.3	12.5
Co-trimoxazole	91.7	75
Linezolid	100	^*∗∗*^

## Data Availability

The data that support the findings of this study are available on request from the corresponding author.
